# Management of a Case of a Large Perforation Repair With Biodentine

**DOI:** 10.7759/cureus.62020

**Published:** 2024-06-09

**Authors:** Tejas Suryawanshi, Manoj Chandak, Aditya Patel, Anuja Ikhar, Shweta Sedani, Namrata P Jidewar, Palak Hirani, Paridhi Agrawal

**Affiliations:** 1 Conservative Dentistry and Endodontics, Sharad Pawar Dental College and Hospital, Datta Meghe Institute of Higher Education and Research, Wardha, IND

**Keywords:** iatrogenic perforation, furcal perforation, endo-perio lesions, biodentine, perforation repair

## Abstract

Perforations, which are artificial contact connections among teeth and supporting tissues, have a substantial impact on the success of root canal therapy, whether caused by iatrogenic or pathological causes. This case report describes a 51-year-old female who had intermittent jaw pain that was diagnosed as perforation and was successfully controlled with endodontic intervention following a referral due to procedural problems. The perforation in the furcation zone of a molar was treated with biodentine, demonstrating its sealing, biocompatibility, and tissue restoration properties. The discussion emphasizes the necessity of choosing the right repair materials and techniques based on perforation size and location. Biodentine emerges as a viable option due to its capacity to form a dependable seal in demanding settings.

The study concluded by emphasizing the need for physician competence, tooth morphology understanding, and operative proficiency in preventing and properly treating perforations for the best treatment outcomes.

## Introduction

Root canal treatments play a pivotal role in preserving dental health by addressing various issues such as pulp infection and necrosis. However, despite advancements in endodontic techniques, complications can arise, potentially jeopardizing treatment success. Among these complications, perforations represent a significant challenge due to their detrimental effects on the integrity of the tooth structure and surrounding tissues. Perforations are artificial contacts between the tooth and supporting tissues that are caused by iatrogenic or pathological processes [1]. Pathological communication is caused by resorption and caries, whereas iatrogenic communication occurs during root canal treatment [2]. Perforations can occur in different locations within the tooth, including furcation areas, root surfaces, and the pulp chamber floor, with causes ranging from iatrogenic errors to pathological processes. Their implications for dental health are profound, often leading to compromised treatment outcomes and the risk of infection or tooth loss if left unaddressed. Prolonging perforation repair can lead to a poor prognosis. Perforations are characterized according to their location: coronal perforations, furcation perforations, post-space perforations, and root canal perforations [3,4]. Current strategies for managing perforations encompass a range of materials and techniques, aiming to achieve effective sealing and restoration of tooth integrity. Recent literature highlights successes with various materials but also underscores limitations, emphasizing the need for innovative solutions. Introducing biodentine as a material for perforation repair; its unique properties, including excellent biocompatibility, bioactivity, and ability to promote cementogenesis, offer promising advantages in overcoming the challenges associated with perforation management. This case report describes the successful management of a furcation perforation in a multi-rooted molar using biodentine, showcasing its efficacy in addressing this challenging clinical scenario and highlighting its relevance in optimizing dental treatment outcomes and minimizing complications.

## Case presentation

A 51-year-old female patient complained of mild intermittent pain in the lower region left back of her jaw, based on the Heft-Parker Visual Analogue Scale, which was associated with sensitivity to hot and cold substances. The pain was spontaneous in nature and notably exacerbated during the night while lying down.

The patient visited a private dental clinic in Higanghat for the same complaint, and an endodontic procedure was initiated but halted due to a procedural complication. The patient was referred to the Department of Conservative Dentistry and Endodontics at Sharad Pawar Dental College and Hospital (SPDC&H) for further management. When reported to SPDC&H, the patient was alleviated of her pain. She had no history of pain with heat or cold stimuli since her last dental visit at Hinganghat. There was no history of suffering from biting or nighttime pain. There was no relevant medical history for systemic pain. There was no relevant dental history available. Clinical temporary restoration with tooth numbers 36 and 35 resulted in no tenderness on percussion.

**Figure 1:**
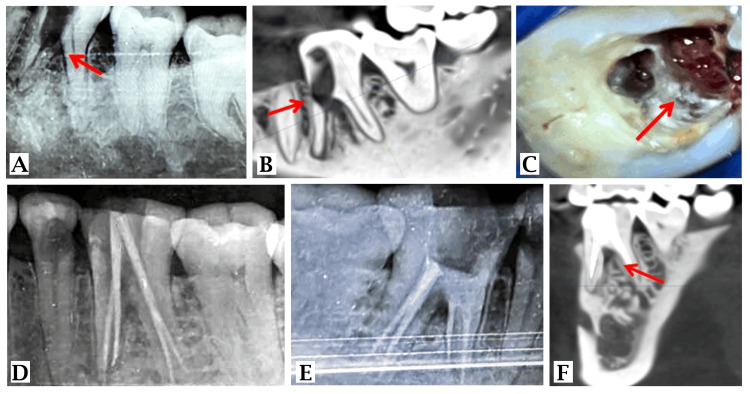
Radiographs and clinical photographs taken preoperatively, intraoperatively, and postoperatively 1A: intra-oral periapical radiograph of 36; 1B: CBCT with 36; 1C: granulation tissue at the perforation site; 1D: mastercone radiograph with 36; 1E: immediate post-operative X-ray after obturation and biodentine placement; 1F: six-month follow-up CBCT CBCT: cone-beam computed tomography

The electric pulp test was performed with tooth numbers 35 and 36. Tooth numbers 35 and 36 exhibited no response when compared to adjacent and contralateral tooth numbers 38 (28), 45 (24), and 46 (26). The preoperative radiograph showed periapical PDL widening with tooth number 35. Radiolucency on the pulpal floor communicating with the periodontium in relation to the mesial root of tooth number 36. Figure 1A shows a mild widening of the periodontal ligament (PDL) space relative to the mesial and distal roots of tooth number 36. Cone-beam computed tomography (CBCT) was recommended for improved fault visualization.

With tooth numbers 35 and 36, the access cavity is clearly visible. Tooth numbers 35 and 36 exhibit a widening of the apical PDL. Pulpal floor discontinuity seen with tooth number 36 (defect size 5.1 mm x 2.6 mm) suggests furcal perforation in tooth number 36's mesial root, as shown in Figure 1B. Final diagnosis: previously initiated root canal treatment, as well as asymptomatic apical periodontitis with tooth numbers 35 and 36. Pulpal floor perforation with tooth number 36.

We administered 2% lidocaine and a 1:80,000 adrenaline ratio. The rubber dam application was completed with tooth numbers 35 and 36. The procedure was performed under a dental operating microscope, and the temporary restoration was removed with tooth number 35. The working length was measured with a Root Zx small apex locator (J. Morita, USA). Biomechanical preparation (BMP) was performed with an F2 6% Hand Protaper (Dentsply, USA). The canal was washed out with 2.5% sodium hypochlorite (NaoCl), normal saline (NS), and 2% chlorhexidine (CHX). Tooth number 35 was given a temporary restoration. After removing the restoration with tooth number 36, unprovoked profuse bleeding occurred with tooth number 36 following the removal of a temporary restoration that was making the negotiation of the canals, as shown in Figure 1C.

A local hemostatic substance was applied to a cotton pellet in an attempt to stop the bleeding. After removing the cotton pellet, the distal canals were visible, and subsequent attempts to locate the mesial canals resulted in more bleeding in the pulp chamber. Disto-lingual and disto-buccal canals were negotiated. The working length was measured using the Root Zx small apex locator (J. Morita, Japan). For tooth number 36, the disto-buccal canal measured 16 mm, while the disto-lingual canal measured 16.5 mm.

Attempts to locate mesial canals failed due to granulation tissue and hemorrhage. The tissue was degranulated in the perforation, and hemostasis of the perforated area was achieved using a liquid hemostat. Mesial canals were negotiated (mesio-buccal=16 mm, mesio-lingual=16 mm), and BMP was performed with an F2 NiTi rotary file (Endo Plus Woodpecker) until 25/6%. Radiographs were used to assess the master cone fit. Following cone fit evaluation, as shown in Figure 1D, tooth number 36 canals were obturated (Ceraseal Bioceramic-based Sealer by Cerkamed), as shown in Figure 1E. 

Following the obturation of all four canals. Biodentine (Septodont) was condensed within the perforated region using a curved mineral trioxide aggregate (MTA) carrier (Angelus, USA). Then a cotton pellet was kept over it, and glass ionomer cement (Ketac Molar, 3M) was given as a temporary restoration. After 24 hours, resin-modified glass ionomer cement (ReGlass by Safe Endo) was placed as the final restoration. The patient was recalled to the department for one month, three months, and six months for follow-up examinations, as shown in Figure 1F. The patient's symptoms improved after her first follow-up visit, including tenderness and pain on biting.

## Discussion

Perforation creates an open route for bacteria to enter, either from the root canal or periodontal tissues, or both, causing an inflammatory reaction that can result in fistulae and bone resorption processes [5]. When a perforation occurs laterally or in the furcation area, the gingival epithelium may overgrow towards the perforation site, compromising the tooth's prognosis [5]. Depending on the perforation's size and location, it can be repaired conservatively or surgically [6].

All perforations must be sealed to prevent the admission of toxic materials from within the tooth, which could create difficulties [6]. The materials that can be used are zinc oxide eugenol, Super EBA, intermediate restorative material (IRM), cavit, glass ionomer cement, composite, dentin chips, decalcified freeze-dried bone, calcium phosphate cement, tricalcium phosphate cement, hydroxyapatite, mineral trioxide aggregate, biodentine, and bioaggregate. Mineral trioxide aggregate demonstrated good treatment outcomes due to its biocompatibility and low tissue toxicity, as it is a time-tested material [7].

Biodentine can form an acceptable seal in the presence of moisture and blood, which is the most significant benefit when employed as a furcation repair material. Biodentine has an alkaline pH of 12.5 and promotes periodontal ligament repair and cementogenesis, combined with a shorter setting time and a higher push-out bond strength. Hence, for this case of furcation perforation, biodentine was used [8,9]. Because there was interaction with the underlying periodontium as well as contamination from blood and moisture, a moisture-resistant substance was required. MTA was found inferior to biodentine in handling properties, biocompatibility, setting time, ion release, and bond strength [10]. With all of these characteristics, biodentine is the most commonly utilized perforation repair material.

A comparable instance involving the successful repair of a substantial perforation with a defect size of less than 2 mm was documented with a follow-up period of four months [11]. In the current case report, the perforation site exceeded 2 mm, nearly bisecting the molar, yet due to meticulous consideration of the physical properties of the utilized material and restorative techniques, a favorable outcome was achieved, as evidenced by a successful follow-up evaluation spanning six months.

## Conclusions

A perforation is an undesirable complication of treatment that can happen to anyone. Regardless of whether the treatment is surgical or non-surgical, several elements can have a substantial impact on the success of the repair. To avoid developing a perforation in the first place, the clinician must have a thorough understanding of tooth morphology, strong clinical judgment, and competent clinical operative abilities. By embracing biodentine as the go-to material for perforation repair, clinicians can confidently address this challenging complication while striving for predictable and successful treatment results. Moreover, ongoing research and larger-scale studies are imperative to further validate the efficacy and benefits of biodentine in perforation management, consolidating its position as a cornerstone material in endodontic practice.
